# Distinct MicroRNA Expression Signatures of Porcine Induced Pluripotent Stem Cells under Mouse and Human ESC Culture Conditions

**DOI:** 10.1371/journal.pone.0158655

**Published:** 2016-07-06

**Authors:** Wei Zhang, Liang Zhong, Jing Wang, Jianyong Han

**Affiliations:** State Key Laboratory for Agrobiotechnology, College of Biological Sciences, China Agricultural University, Beijing, China; Huazhong Agricultural University, CHINA

## Abstract

It is well known that microRNAs play a very important role in regulating reprogramming, pluripotency and cell fate decisions. Porcine induced pluripotent stem cells (piPSCs) are now available for studying the pluripotent regulation network in pigs. Two types of piPSCs have been derived from human and mouse embryonic stem cell (ESC) culture conditions: hpiPSCs and mpiPSCs, respectively. The hpiPSCs were morphologically similar to human ESCs, and the mpiPSCs resembled mouse ESCs. However, our current understanding of the role of microRNAs in the development of piPSCs is still very limited. Here, we performed small RNA sequencing to profile the miRNA expression in porcine fibroblasts (pEFs), hpiPSCs and mpiPSCs. There were 22 differential expressed (DE) miRNAs down-regulated in both types of piPSCs compared with pEFs, such as ssc-miR-145-5p and ssc-miR-98. There were 27 DE miRNAs up-regulated in both types of piPSCs compared with pEFs. Among these up-regulated DE miRNAs in piPSCs, ssc-miR-217, ssc-miR-216, ssc-miR-142-5p, ssc-miR-182, ssc-miR-183 and ssc-miR-96-5p have much higher expression levels in mpiPSCs, while ssc-miR-106a, ssc-miR-363, ssc-miR-146b, ssc-miR-195, ssc-miR-497, ssc-miR-935 and ssc-miR-20b highly expressed in hpiPSCs. Quantitative stem-loop RT-PCR was performed to confirm selected DE miRNAs expression levels. The results were consistent with small RNA sequencing. Different expression patterns were observed for key miRNA clusters, such as the miR-17-92 cluster, the let-7 family, the miR-106a-363 cluster and the miR-182-183 cluster, in the mpiPSCs and hpiPSCs. Novel miRNAs were also predicted in this study, including a putative porcine miR-302 cluster: ssc_38503, ssc_38503 and ssc_38501 (which resemble human miR-302a and miR-302b) found in both types of piPSCs. The miR-106a-363 cluster and putative miR-302 cluster increased the reprogramming efficiency of pEFs. The study revealed significant differences in the miRNA signatures of hpiPSCs and mpiPSCs under different pluripotent states that were derived from different culture conditions. These differentially expressed miRNAs may play important roles in pluripotent regulation in pigs, and this information will facilitate the understanding of the mechanism of pluripotency in pigs.

## Introduction

The pig is an excellent model for human disease because of its similarity to humans in terms of its organ morphology and function. However, no authentic porcine embryonic stem cells (pESCs) have been isolated successfully. The mechanisms of porcine early embryonic development and the establishment of a pluripotent regulation network have been largely unclear. Porcine induced pluripotent stem cells (piPSCs) now become an excellent alternative resource of pESCs, which have potential application prospect in regeneration medicine and cell therapy [1–4]. The piPSCs also can provide researchers with useful tools for studying the regulation of pluripotency in pigs [5].

MicroRNAs, which have 20 to 22 nucleotides RNA sequences, play a major role in post-transcriptional gene regulation in higher eukaryotes. The miRNAs usually specifically bind to partially complementary target 3’ UTR regions of mRNAs to cause gene silencing via translational repression or target degradation [6]. It was recently reported that microRNAs can also bind the CDS region to regulate gene expression [7]. Families of microRNAs with highly conserved sequences are found in many organisms, and clusters of miRNAs located in discrete genomic loci can be coordinately expressed. The clusters and families of miRNAs are ideal molecular tools for regulating developmental processes, as these miRNAs can be expressed in a temporal manner to regulate the expression of a host of genes [8]. Studies have also shown that miRNAs and miRNA clusters play critical roles in the reprogramming process and maintenance of pluripotent stem cells in humans and mice. MicroRNA clusters such as the miR-302-367 cluster and miR-290-295 cluster are very important to maintaining the self-renewal and pluripotency of mouse embryonic stem cells (mESCs) [9–11]. Naïve and primed murine pluripotent stem cells have distinct miRNA expression profiles [12]. Human embryonic stem cells (hESCs) have a special miRNA expression pattern that differs from that of mESCs [13,14]. The primed hESCs can be converted to a naïve state following a change in miRNA expression [15].

MiRNAs also improve the efficiency of iPSCs induction. For example, expression of the miR-302-367 cluster was shown to be sufficient to drive the efficient reprogramming of murine and human somatic cells to a pluripotent state [16–18]. Similarly, the miR-200 family inhibits the TGF-β signaling pathway to promote reprogramming [19]. High-quality mouse iPSCs have active miRNA expression in the Dlk1-Dio3 region and are capable of producing full-term mice through tetraploid complementation [20,21]. These previous findings all indicate that miRNAs not only are very important to maintain pluripotency and regulate the reprogramming process but also have distinct expression patterns under different stem cell states.

Using defined hESC medium and mESC medium, we have previously induced two types of piPSCs: hpiPSCs and mpiPSCs. These cells have different characteristics in terms of their pluripotent markers, metabolism and gene expression [22]. However, the changes in the miRNA profiles associated with the modulation of pluripotency and self-renewal in these two types of piPSCs are largely unknown. Identifying these two types of piPSCs miRNA profiles will be an important step toward understanding the functional involvement of miRNAs in the induction of pluripotency and the effects of different culture conditions on the miRNA expression pattern. With this in mind, we aimed to use small RNA sequencing to identify the miRNA profiles of porcine embryonic fibroblasts (pEFs), hpiPSCs and mpiPSCs and to further characterize the key miRNAs involved in establishing pluripotency, as well as to identify the putative pathways targeted by the miRNAs in this study.

## Methods and Materials

### Cell culture

The cell lines used in this study: pEFs, hpiPSCs and mpiPSCs were established in our lab and reported in our former study [22]. Briefly, the pEFs were cultured in DMEM containing 10% fetal bovine serum (Gibco, 10099–141), 1% MEM non-essential amino acid (Gibco, 11140), 1% GlutaMAX (Gibco, 35050), and 1% penicillin/streptomycin (Gibco, 15140). The two kinds of piPSC lines were cultured under different culture conditions, with the hpiPSCs cultured in commercial hESC culture medium (NutriStem XF/XX medium, Stemgent 01–0005). The mpiPSCs were cultured in DMEM containing 10 μg/L human LIF (Millipore, LIF1005), 3 μM CHIR99021 (Selleck, S1036), 2 μM PD035021 (Selleck, 252917), 1% MEM non-essential amino acid (NEAA), 1% GlutaMAX, 1% penicillin/streptomycin, and 1% sodium pyruvate (Gibco, 11360). Both types of piPSCs were cultured on mitomycin C inactivated mouse embryonic fibroblast-coated plates.

### Alkaline phosphatase (AP) staining

The alkaline phosphatase (AP) activity of the piPSCs was determined using an Alkaline Phosphatase Detection Kit (Millipore, SCR004) according to the manufacturer’s instructions.

### RNA extraction

Small RNAs from the piPSCs and pEFs were extracted using the miRNeasy Mini Kit (QIAGEN, 217004) following the manufacturer’s recommendations. The RNA samples were examined by measuring the OD260/280 ratio. Samples with a ratio of 2.0 were used for experiments.

### Library construction and sequencing

The small RNA digitalization analysis based on HiSeq high-throughput sequencing uses SBS (sequencing by synthesis), which can decrease the loss of nucleotides caused by the secondary structure. Small RNA sequencing raw-reads were generated from two biological replicates each from the fibroblasts, hpiPSCs and mpiPSCs. Libraries for Illumina deep sequencing were prepared using the NEB Multiplex Small RNA Library Prep Set for Illumina according to the manufacturer’s description, and libraries were sequenced on the Illumina HiSeq 2500. This work was performed at the Beijing Genomics Institute (BGI), Beijing, China.

### Small RNA sequencing analysis

The raw reads were aligned to the genome (Sscrofa10.2/susScr3) with Bowtie2 software. The miRNA counts were estimated using the Htseq-0.6.0 program and were normalized by calculating the tags per million (TPM). The microRNA annotation database used was miRBase v21. To identify the short sequences, we used the BLAST+ and rfam database v11 software to make classifications for the major non-coding RNA category [23]. A heatmap of small RNA profiles related to the Z-score of the TPM value was generated for each microRNA, and the correlation heatmap was associated with the matrix of Pearson correlation coefficient values between each of the samples. The significance of the differentially expressed miRNAs was analyzed using DEseq2 as an R/Bioconductor package [24]. A statistical test with Benjiamini and Hochberg false discovery rate (BH FDR) was performed to adjust the P-value. Finally, |log_2_(FC)| ≥1, total counts >10 and adjusted P-value <0.05, were set as the threshold for selection of differentially expressed (DE) miRNAs.

### Prediction of novel porcine miRNAs by miRDeep2

To identify novel miRNAs from the small RNA libraries, we used the miRDeep2 software package [25]. The unique sequences were mapped to the *Sus Scrofa 10*.*2* reference genome with the read aligner (Bowtie2; mapper module), allowing only alignments with 0 mismatch in the first 18 nt of a read sequence and up to two mismatches after 18 nt and keeping unique reads that did not map more than five times to the genome. For miRNA prediction with the core algorithm of miRDeep2, both mouse and human mature miRNAs, including previously reported *Sus Scrofa* mature miRNAs and hairpins (both retrieved from miRBase 21), along with mapped reads from the previous step, were used as input. The initial miRDeep2 output list of candidate miRNA precursors from each library was manually curated to generate a final high-confidence set of miRNAs retaining only candidate novel precursors. The parameters used to screen for novel miRNAs predicted using miRDeep2 were as follows: (a) miRDeep2 score >100; (b) the ratio of mature miRNA vs pre-miRNA; and (c) only a 2 nucleotides overhang for the hairpin structure, which is the hallmark of a bona fide miRNA.

### Target gene prediction and functional analysis

The target genes of the differentially expressed miRNAs were predicted using the miRWalk target prediction software [26,27]. In this software program, the common target genes that were predicted by different algorithms, such as miRWalk, miRanda [28], miRDB [29], RNA22 [30] and Targetscan [31], were chosen. Because porcine genes were not included in the current version of miRWalk, predictions were performed using human miRNAs. Only conserved miRNAs and conserved targets were included. A functional analysis of the predicted target genes was performing using the *Gostats* package to perform the annotation of the Gene Ontology (GO) and KEGG pathway. Hypergeometric test was used to classify the GO category and KEGG pathway analysis. The resulting P-values were adjusted using the BH FDR algorithm. GO and KEGG pathway categories with adjusted P-value < 0.05 were reported.

The DIANA miRPath software was used to perform an enrichment analysis of the miRNA cluster target genes comparing each set of microRNA targets to all known KEGG pathways [32]. Because porcine genes were not included in the current version of the DIANA miRPath software, the prediction was performed using conserved human miRNAs. Signaling pathways with adjusted P-value <0.05 were selected.

### Quantitative stem-loop RT-PCR assay of the differentially expressed miRNAs

To validate the miRNAs identified by small RNA sequencing, a stem-loop reverse-transcription quantitative polymerase chain reaction (RT-PCR) assay was used to specifically detect mature miRNAs. The stem-loop RT-PCR primers were designed according to the previously reported methods [33,34]. The samples for quantitative stem-loop RT-PCR were same as sequencing samples. 2 μg RNA of each sample was used for stem-loop RT-PCR. The reverse transcription was performed using specifically stem-loop RT-PCR primers and M-MLV Reverse Transcriptase (Promega, M1701). U6 was used as the endogenous control. The quantitative stem-loop RT-PCR analysis was performed using SYBR Green I PCR Master Mix (Roche, 4887352001), and the products were detected with the Light cycler 480 II (Roche). The relative quantification of miRNA expression was performed using the comparative CT (2^-△△CT^) method. The data were presented as the mean ± SD of triplicated wells. The Student’s t-test was used to analyze the expression difference between the 2 groups. The primer sequences used are listed in S1 Table.

### Construction of miRNA cluster expression vectors

Pig genomic DNA was extracted from pig embryonic fibroblasts (pEFs) using a QIAamp DNA Mini Kit (QIAGEN, 51304) according to the manufacturer’s instructions. The pig genomic DNA fragments comprising miRNA clusters were amplified by PCR using TransStart FastPfu DNA Polymerase (TransGen Biotech, J10203). PCR was performed under the following conditions: 95°C for 5 min, 35 cycles of denaturation at 95°C for 20 s, annealing at 60°C for 20 s, elongation at 72°C (1 kb/min), and a final extension at 72°C for 10 min. The amplified fragments were cloned into a retroviral pMXs vector (laboratory stored) and verified by sequencing. The primer sequences used for amplification are listed in S1 Table.

### Retrovirus production and piPSCs induction

The pMXs system separately carrying the porcine miR-106a-363 cluster and putative porcine miR-302 cluster were used to reprogram the pEFs in combination with pMXs-Oct4, pMXs-Sox2, pMXs-Klf4 and pMXs-Myc (OSKM, laboratory stored). The pMXs-GFP plus OSKM were used as a control group. Virus production was performed using a previously described method [22]. Briefly, retroviruses were used to infect pEFs for 12 h in the presence of 8 μg/ml polybrene (Sigma, 107689). After two rounds of infection, the infected cells were passaged and seeded into feeder plates, followed by a change in the reprogramming medium on the second day. The reprogramming medium was DMEM containing 10 μg/L human LIF, 10% Knockout Serum Replacement (Gibco, 10828–028), 1% NEAA, 1% GlutaMAX, 1% sodium pyruvate, and 1% penicillin/streptomycin. The medium was changed every two days. The reprogramming efficiency was evaluated by AP staining on day 10.

## Results

### Different miRNA expression profiles of pEFs, hpiPSCs and mpiPSCs

**Fig 1:**
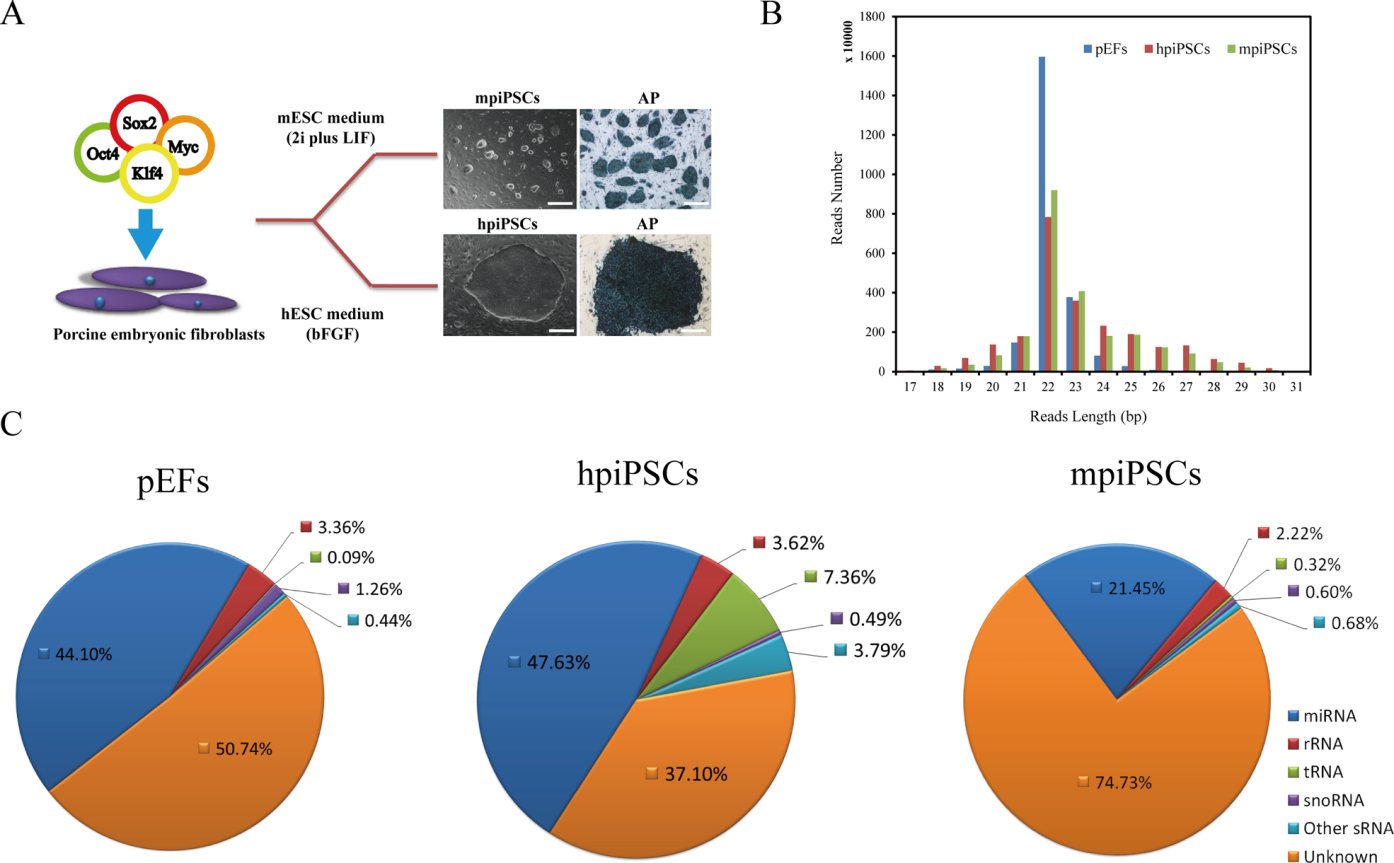
The distribution of small RNA reads and various RNA classes in piPSCs and pEFs. (A) The morphological features and AP staining of piPSCs. Scale bar = 500 μm. (B) The distribution of small RNA reads in the piPSCs and pEFs. (C) Pie chart showing the various RNA classes in pEFs and piPSCs.

**Fig 2:**
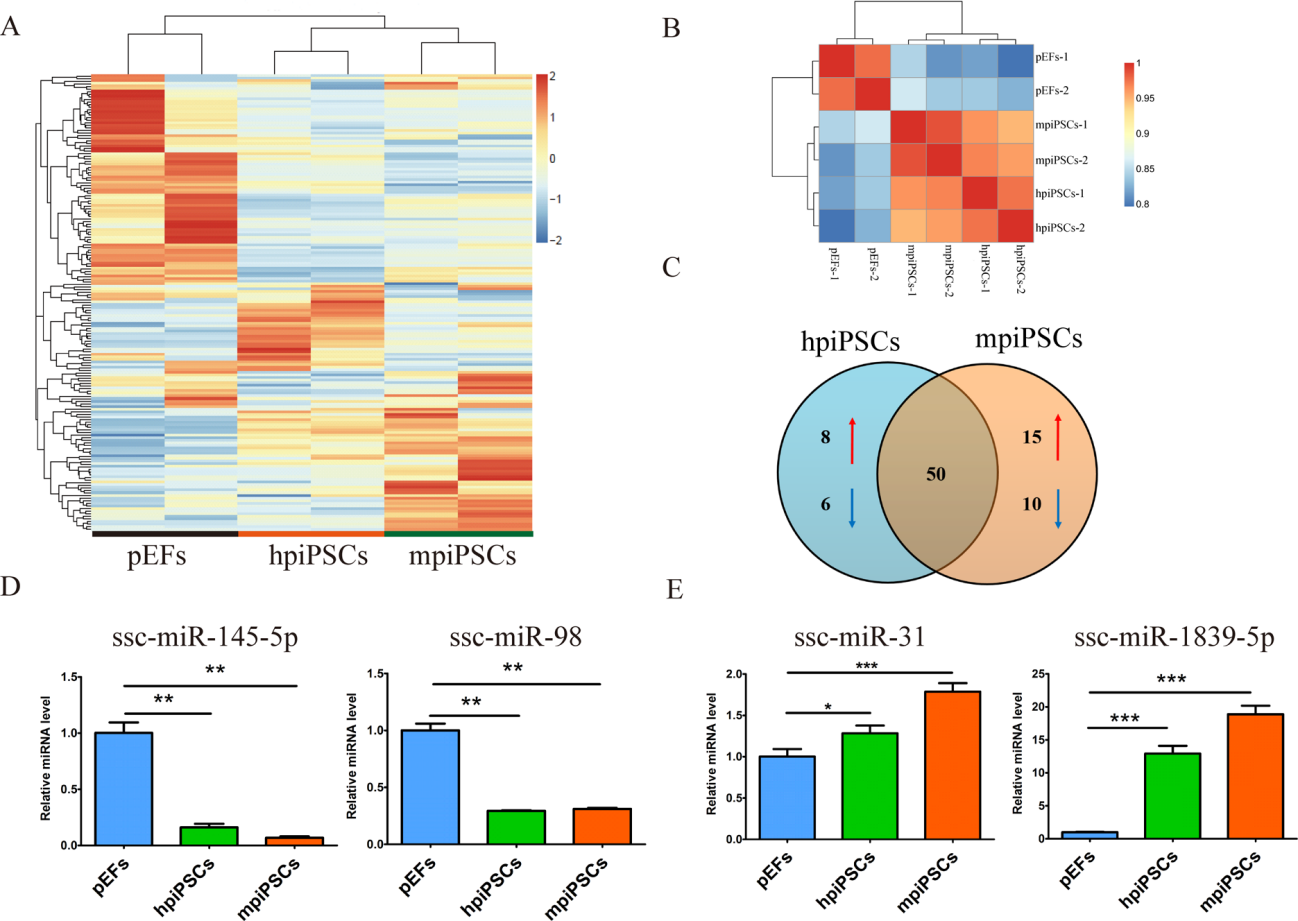
MicroRNA expression profiles of piPSCs and pEFs. (A) Heatmap of the microRNAs profiles in piPSCs and pEFs. (B) Pearson correlation coefficients of the overall microRNA expression of piPSCs and pEFs. (C) Venn diagram of the differentially expressed microRNAs in the piPSCs compared with the pEFs. (D) Results of a quantitative stem-loop RT-PCR assay of the ssc- miR-98 and ssc-miR-145-5p expression in pEFs, hpiPSCs and mpiPSCs. Mean values ± SD are shown. *P-value <0.05, ***P-value < 0.001. (E) Results of a quantitative stem-loop RT-PCR assay of ssc-miR-1839-5p and ssc-miR-31 in pEFs, hpiPSCs and mpiPSCs.

We previously reported that two types of piPSCs were derived from different culture conditions [22]. The hpiPSCs cultured in hESC medium (containing bFGF) were found to have a hESC-like state or epiblast-type morphology, and the colonies were flat and large. The mpiPSCs maintained in 2i plus LIF medium were similar to mESCs in terms of their morphology. They were three-dimensional and rounded. The two types of cells, mpiPSCs and hpiPSCs, were both positive for AP staining (**Fig 1A**). To investigate the miRNA profiles of pEFs and piPSCs, we performed small RNA sequencing, which ranged from 16 to 20 million reads (**S1 File**). Based on the clean reads, the pEFs, hpiPSCs and mpiPSCs followed the typical read distribution for small RNA sequencing, with the majority of raw reads belonging to the mature miRNA range of 22–23 nucleotides (**Fig 1B**). Based on the total annotated reads, various classes of small RNAs were distributed in the pEFs and two types of piPSCs. Among these small RNAs, the known mature miRNAs comprised 21.45% of the sequences in mpiPSCs, 47.63% in hpiPSCs and 44.10% in pEFs (**Fig 1C**). A hierarchical cluster analysis of the miRNA profiles showed that the mpiPSCs clustered with hpiPSCs, and both were distant from the pEFs (**Fig 2A** and **Fig 2B**). Venn diagram showed the numbers of differential expressed (DE) miRNAs in piPSCs compared with pEFs (**Fig 2C**). Compared with pEFs, there were 50 DE miRNAs in both types of piPSCs. Among these DE miRNAs, there 22 were down-regulated in both types of piPSCs compared with pEFs (**Table 1**), while 27 DE miRNAs up-regulated in both types of piPSCs versus pEFs (**Table 2**).14 DE miRNAs specifically differential expressed in hpiPSCs versus pEFs, while 25 miRNAs specifically differential expressed in mpiPSCs versus pEFs. To validate the differential expression identified by the miRNA sequencing, ssc-miR-145-5p, ssc-miR-98, ssc-miR-31 and ssc-miR-1839-5p were selected for quantitative stem-loop RT-PCR analysis. Compared with pEFs, expression of ssc-miR-145-5p and ssc-miR-98 were down-regulated in both two types of piPSCs (**Fig 2D**), whereas ssc-miR-1839-5p and ssc-miR-31 were both up-regulated in both types of piPSCs (**Fig 2E**).

**Table 1:** Differentially expressed miRNAs down-regulated in both two types of piPSCs compared with pEFs (|log_2_(FC)|≥1, total counts >10, P-value < 0.05).

	mpiPSCs versus pEFs			hpiPSCs versus pEFs		
miRNAs	log_2_(FC)	P-value	P-value.adj	log_2_(FC)	P-value	P-value.adj
ssc-miR-21	-1.1788	1.45E-02	1.68E-02	-2.4642	2.07E-04	3.85E-04
ssc-miR-143-3p	-1.1940	1.40E-02	1.67E-02	-2.7004	2.27E-05	5.34E-05
ssc-miR-145-3p	-1.2289	2.47E-02	2.68E-02	-2.6837	6.34E-04	1.10E-03
ssc-miR-505	-1.3657	2.68E-02	2.82E-02	-2.1577	4.16E-02	4.16E-02
ssc-miR-98	-1.5185	3.46E-03	5.15E-03	-2.8061	7.55E-05	1.55E-04
ssc-miR-139-3p	-1.6685	2.54E-02	2.71E-02	-2.5158	1.69E-02	1.93E-02
ssc-miR-23b	-1.7157	3.70E-03	5.42E-03	-2.3687	8.39E-03	1.10E-02
ssc-miR-224	-1.8515	1.41E-02	1.67E-02	-2.5778	1.95E-02	2.19E-02
ssc-miR-23a	-1.8753	3.40E-03	5.15E-03	-2.4676	1.00E-02	1.24E-02
ssc-miR-143-5p	-1.9243	1.15E-04	2.60E-04	-3.9943	1.25E-09	5.88E-09
ssc-miR-139-5p	-2.1198	2.01E-02	2.24E-02	-3.2644	1.01E-02	1.24E-02
ssc-miR-222	-2.2666	2.58E-07	1.02E-06	-2.6019	2.34E-05	5.35E-05
ssc-miR-671-5p	-2.3068	1.15E-02	1.47E-02	-2.7986	3.86E-02	3.92E-02
ssc-miR-9843-3p	-2.3507	9.68E-04	1.87E-03	-4.7281	5.90E-05	1.31E-04
ssc-miR-145-5p	-2.7059	2.08E-03	3.50E-03	-4.3459	7.18E-05	1.51E-04
ssc-miR-221-5p	-2.7136	3.21E-07	1.21E-06	-1.9513	3.02E-02	3.22E-02
ssc-miR-221-3p	-2.9643	8.31E-11	5.47E-10	-2.1967	1.74E-03	2.90E-03
ssc-miR-708-5p	-4.0615	2.31E-06	7.60E-06	-2.8238	6.43E-03	8.72E-03
ssc-miR-193a-3p	-4.1933	2.39E-07	1.02E-06	-4.3848	2.87E-07	9.18E-07
ssc-miR-193a-5p	-4.1933	2.39E-07	1.02E-06	-7.1423	2.32E-12	1.33E-11
ssc-miR-452	-4.3025	5.55E-11	3.99E-10	-2.2057	1.53E-02	1.77E-02
ssc-miR-206	-5.3001	6.39E-09	3.37E-08	-6.2200	3.10E-09	1.38E-08

**Table 2:** Differential expressed miRNAs up-regulated in both two types of piPSCs compared with pEFs (|log_2_(FC)|≥1, total counts >10, P-value < 0.05).

	mpiPSCs versus pEFs			hpiPSCs versus pEFs		
miRNAs	log_2_(FC)	P-value	P-value.adj	log_2_(FC)	P-value	P-value.adj
ssc-miR-216	8.4214	5.03E-07	1.73E-06	5.7690	1.36E-02	1.62E-02
ssc-miR-217	8.1614	4.22E-35	3.33E-33	5.6463	8.33E-24	1.67E-22
ssc-miR-183	7.4376	1.86E-17	2.94E-16	4.2323	9.27E-09	3.71E-08
ssc-miR-182	6.5622	7.25E-17	9.55E-16	3.5249	2.00E-08	7.27E-08
ssc-miR-96-5p	6.0732	1.15E-12	1.30E-11	2.9388	1.57E-04	3.14E-04
ssc-miR-34c	5.1209	4.58E-26	1.81E-24	4.6746	7.23E-28	1.93E-26
ssc-miR-1839-5p	4.6949	2.62E-22	6.90E-21	3.5245	2.11E-19	2.81E-18
ssc-miR-142-5p	4.5982	2.21E-09	1.25E-08	2.1800	2.63E-03	4.07E-03
ssc-miR-18b	4.3294	7.03E-03	9.74E-03	5.0527	1.45E-05	3.74E-05
ssc-miR-101	3.1742	4.51E-06	1.27E-05	1.8970	6.56E-04	1.12E-03
ssc-miR-146b	3.1490	3.90E-10	2.37E-09	5.7777	2.78E-28	1.11E-26
ssc-miR-363	3.1237	4.35E-12	4.30E-11	3.5880	4.91E-20	7.86E-19
ssc-miR-106a	3.0330	3.87E-05	9.86E-05	3.8702	2.13E-11	1.14E-10
ssc-miR-378	3.0141	2.52E-07	1.02E-06	2.6676	1.00E-08	3.81E-08
ssc-miR-2483	2.9709	2.75E-06	8.69E-06	1.8246	6.20E-05	1.34E-04
ssc-miR-20b	2.9705	3.79E-06	1.15E-05	2.9705	3.79E-06	1.15E-05
ssc-miR-7139-3p	2.7450	1.59E-08	7.85E-08	2.5943	2.34E-11	1.17E-10
ssc-miR-7139-5p	2.6714	5.75E-05	1.38E-04	2.2454	1.68E-05	4.12E-05
ssc-miR-192	2.5572	3.88E-08	1.80E-07	1.5053	1.25E-06	3.70E-06
ssc-miR-345-5p	2.4725	1.25E-03	2.33E-03	1.6975	3.59E-03	5.13E-03
ssc-miR-497	2.4609	4.50E-05	1.11E-04	3.5608	5.54E-13	4.03E-12
ssc-miR-31	2.2685	4.34E-07	1.56E-06	1.2385	4.17E-06	1.15E-05
ssc-miR-194a	2.2010	2.22E-03	3.65E-03	1.3155	9.59E-03	1.22E-02
ssc-miR-195	1.9330	6.81E-04	1.38E-03	3.3227	1.79E-12	1.19E-11
ssc-miR-92b-5p	1.8321	1.77E-05	4.66E-05	2.4872	1.94E-12	1.19E-11
ssc-miR-935	1.7364	3.63E-02	3.63E-02	4.8669	9.23E-08	3.21E-07
ssc-let-7a	1.2422	7.43E-04	1.47E-03	1.1162	1.02E-06	3.14E-06

**Fig 3:**
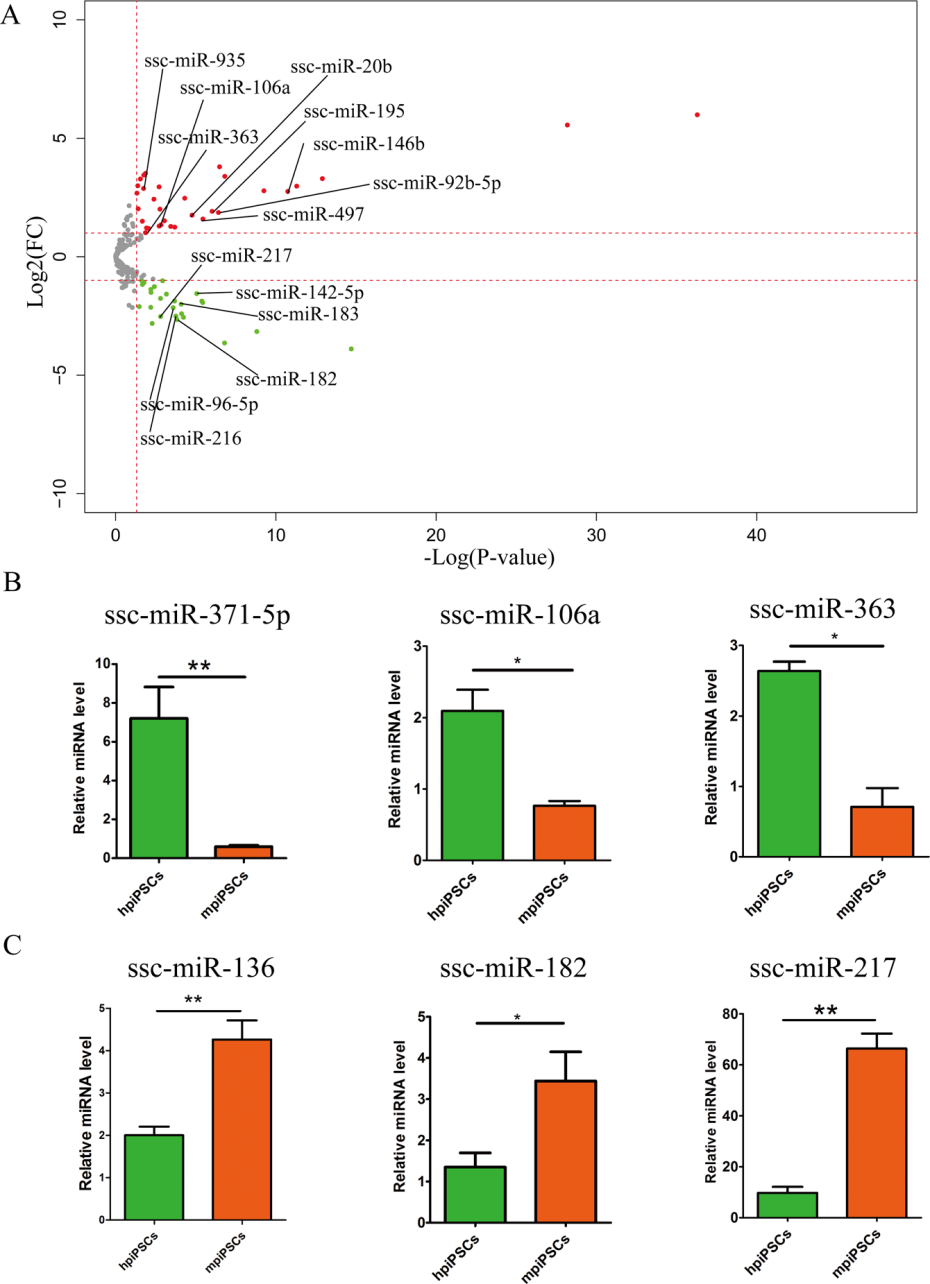
The specific DE miRNAs of hpiPSCs and mpiPSCs. (A) Volcano plot of hpiPSCs versus mpiPSCs. Red plots indicate DE miRNAs up-regulated in hpiPSCs. Green plots indicated DE miRNAs down-regulated in hpiPSCs. DE miRNAs were selected by |log_2_(FC)|≥1, P-value <0.05. (B) Relative expression of selected hpiPSCs-specific miRNAs. (C) Relative expression of selected mpiPSCs-specific miRNAs.

To further explore the differences between the hpiPSCs and mpiPSCs, we found that 15 DE miRNAs were specifically up-regulated in mpiPSCs (**Table 3**), while 8 DE miRNAs were specifically up-regulated in hpiPSCs compared with pEFs (**Table 4**). A direction comparison of hpiPSCs between mpiPSCs was performed (**Fig 3A**). There were 60 miRNAs differentially expressed a criteria of at least 1.5 fold change and adjusted P-value <0.05 (**S2 File**). A series of miRNAs which significantly up-regulated in both two types of piPSCs compared with pEFs have different expression levels in hpiPSCs and mpiPSCs. Ssc-miR-106a, ssc-miR-363, ssc-miR-195, ssc-miR-497, ssc-miR-146b, ssc-miR-92b-5p, ssc-miR-20b and ssc-miR-935 were highly expressed in hpiPSCs than that in mpiPSCs (**Fig 3A**). These 8 miRNAs plus 8 specifically up-regulated miRNAs in hpiPSCs were called hpiPSCs- specific miRNAs. Additionally, ssc-miR-216, ssc-miR-217, ssc-miR-142-5p, ssc-miR-96-5p, ssc-miR-182 and ssc-miR-183 have higher expression levels in mpiPSCs than that in hpiPSCs (**Fig 3A**). These 6 miRNAs plus 15 specifically up-regulated miRNAs in mpiPSCs compared with pEFs were called mpiPSCs- specific miRNAs.

**Table 3:** Differentially expressed miRNAs specifically up-regulated in mpiPSCs versus pEFs (|log_2_(FC)|≥1, total counts >10, P-value < 0.05).

miRNAs	log_2_(FC)	P-value	P-value.adj
ssc-miR-187	6.4779	2.78E-11	2.20E-10
ssc-miR-504	2.6471	9.51E-05	2.21E-04
ssc-miR-10b	2.3151	4.50E-06	1.27E-05
ssc-miR-9810-3p	1.9553	7.28E-03	9.91E-03
ssc-miR-7857-3p	1.7899	3.85E-04	8.44E-04
ssc-miR-331-3p	1.6198	4.56E-04	9.73E-04
ssc-miR-744	1.6136	1.28E-05	3.49E-05
ssc-miR-199a-3p	1.4545	1.27E-03	2.33E-03
ssc-miR-7137-5p	1.3956	3.80E-03	5.46E-03
ssc-miR-136	1.3266	9.49E-03	1.25E-02
ssc-miR-210	1.2791	7.41E-03	9.92E-03
ssc-miR-423-5p	1.1957	1.45E-03	2.61E-03
ssc-miR-103	1.1641	1.26E-02	1.55E-02
ssc-miR-296-3p	1.1181	1.74E-03	3.06E-03
ssc-miR-1306-3p	1.0880	1.42E-02	1.67E-02

**Table 4:** Differentially expressed miRNAs specifically up-regulated in hpiPSCs versus pEFs (|log_2_(FC)|≥1, total counts >10, P-value < 0.05).

miRNAs	log_2_(FC)	P-value	P-value.adj
ssc-miR-371-5p	11.3640	6.94E-19	7.93E-18
ssc-miR-219b-3p	10.1953	2.42E-32	1.94E-30
ssc-miR-218b	5.3242	5.95E-18	5.95E-17
ssc-miR-92b-3p	3.2034	3.39E-17	3.01E-16
ssc-miR-7138-3p	2.0714	1.31E-02	1.59E-02
ssc-miR-219a	2.0675	1.31E-07	4.37E-07
ssc-miR-99a	1.4504	2.83E-06	8.09E-06
ssc-miR-128	1.1854	1.31E-05	3.49E-05

To validate this differential miRNA expression pattern, we performed quantitative stem-loop RT-PCR to assess the expression of the three[35] selected hpiPSCs- specific miRNAs: ssc-miR-371-5p, ssc-miR-106a and ssc-miR-363, which were found to be more highly expressed in hpiPSCs (**Fig 3B**). We also assessed another three selected miRNAs, ssc-miR-136, ssc-miR-217 and ssc-miR-182, which were found to be more highly expressed in mpiPSCs (**Fig 3C**). These results were same with small RNA sequencing.

### Prediction of target genes and the GO and KEGG pathway analyses

**Fig 4:**
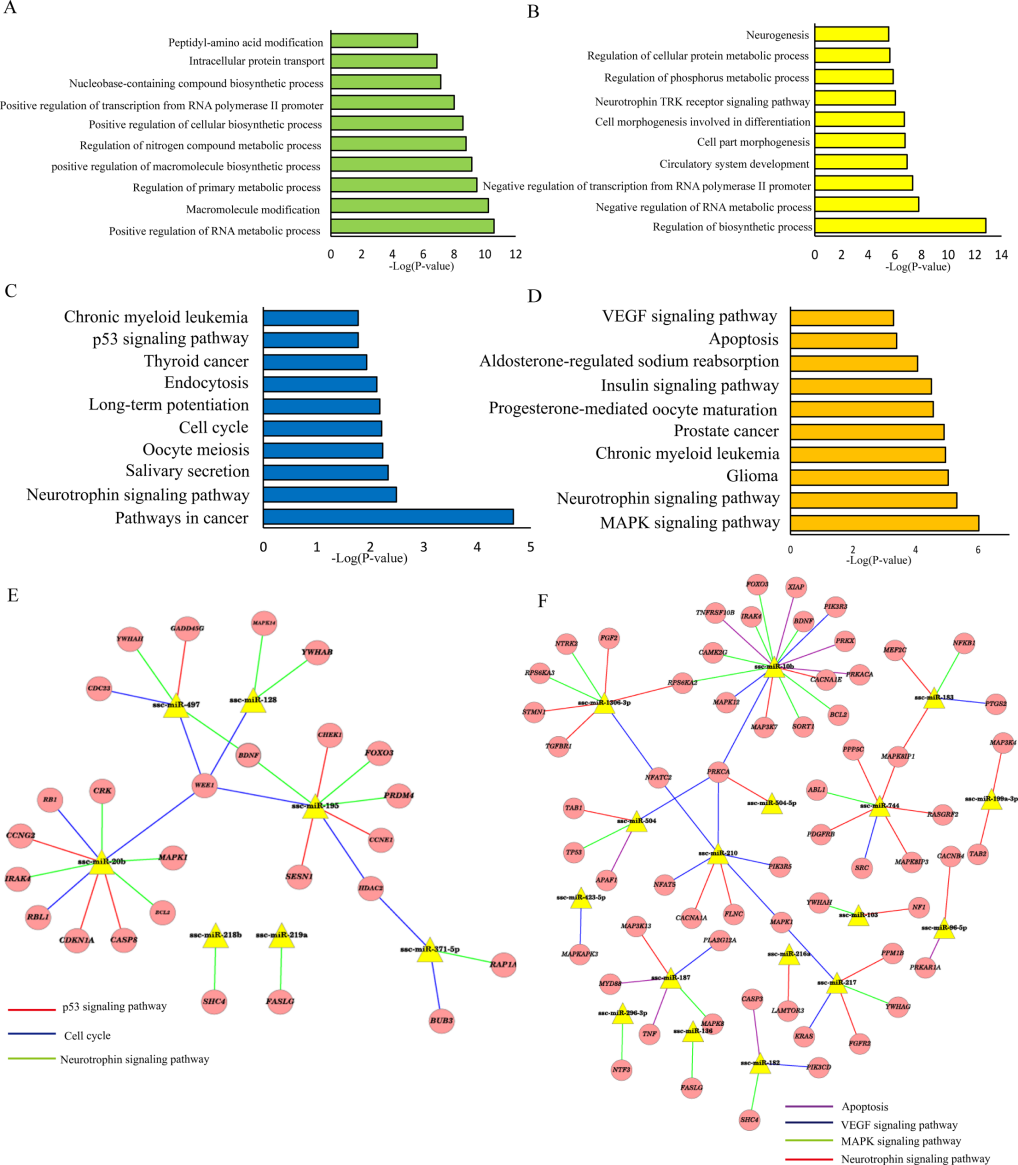
The results of the GO and KEGG analyses of predicted target genes of the differentially expressed miRNAs. (A) The results of a GO analysis of the target genes of hpiPSCs-specific miRNAs (B) The results of a GO analysis of the target genes of mpiPSCs-specific miRNAs. (C) The results of a KEGG pathway analysis of the target genes of hpiPSCs-specific miRNAs. (D) The results of a KEGG pathway analysis of the target genes of mpiPSCs-specific miRNAs. (E) The network of putative target genes and hpiPSCs-specific miRNAs interacting in hpiPSCs. (F) The network of putative target genes and mpiPSCs-specific miRNAs interacting in mpiPSCs.

Despite the limited understanding of the function of many miRNAs, miRNA target prediction programs can identify mRNAs that may be regulated by individual miRNAs. In order to better understand the biological functions of DE miRNAs identified, we therefore combined our present findings with human data using the miRWalk target prediction method. The merged data contained 1459 predicted target genes for 16 hpiPSCs-specific miRNAs and 2001 putative target genes for the 21 mpiPSCs-specific miRNAs (**S3 File**). The predicted target genes were further classified to identify the GO and signaling pathways that were actively regulated by miRNAs in piPSCs according to the GO and KEGG functional annotations. The GO analysis of putative target genes regulated by hpiPSCs-specific miRNAs is shown in **Fig 4A**. Most of the putative target genes were related to positive regulation of RNA metabolic process, macromolecule modification, regulation of primary metabolic process and so on. The GO annotation reflected the regulatory roles that the mpiPSCs-specific miRNAs play in biological processes, including regulation of biosynthetic process, negative regulation of RNA metabolic process, negative, circulatory system development, cell morphogenesis involved in differentiation, neurogenesis (**Fig 4B**). Furthermore, the predicted target genes regulated by hpiPSCs-specific miRNAs were analyzed by KEGG signaling pathways analysis, and the significant pathways (adjusted P-value <0.05) are summarized in **Fig 4C**, including Neurotrophin signaling pathway, cell cycle, and p53 signaling pathway. The results of the pathway analysis of the predicted target genes regulated by mpiPSCs-specific miRNAs are shown in **Fig 4D**. The putative target genes were specifically involved in regulating the MAPK signaling pathway, apoptosis and VEGF signaling pathway and so on. The predicted target genes of the differentially expressed miRNA suggest that these miRNAs may play an important regulatory role in maintaining the pluripotent state of piPSCs. Cytoscape v3.0.1 was used to integrate a potential network of putative target genes and DE miRNAs interacting in hpiPSCs (**Fig 4E**). P53 signaling pathway was regulated by ssc-miR-20b, ssc-miR-497 and ssc-miR-195 through targeting CCNG2, CDKN1A, CASP8, GADD45G, CHEK1, SESN1 and CCNE1. Cell cycle and Neurotrophin signaling pathway were regulated by ssc-miR-20b, ssc-miR-128, ssc-miR-497, ssc-miR-195 and ssc-miR-371-5p through corresponding putative target genes. It suggested that hpiPSCs-specific miRNAs regulated Neurotrophin signaling pathway, cell cycle and p53 pathway to maintain its state. The network of mpiPSCs-specific miRNAs and its predicted target genes was shown in **Fig 4F**. Ssc-miR-182, ssc-miR-187, ssc-miR-136, ssc-miR-210, ssc-miR-217 and ssc-miR-10b participate in regulation Neurotrophin signaling pathway by targeting corresponding genes, including BNDF, SHC4, KRAS and FOXO3. These mpiPSCs-specific miRNAs also were predicted to inhibit VEGF signaling pathway, apoptosis and MAPK signaling pathway through putative target genes. The prediction suggested that mpiPSCs-specific miRNAs may maintain its naïve-like state by regulation Neurotrophin signaling pathway, apoptosis, VEGF signaling pathway and MAPK signaling pathway.

### Two types of piPSCs express different sets of pluripotency-associated miRNA clusters

**Fig 5:**
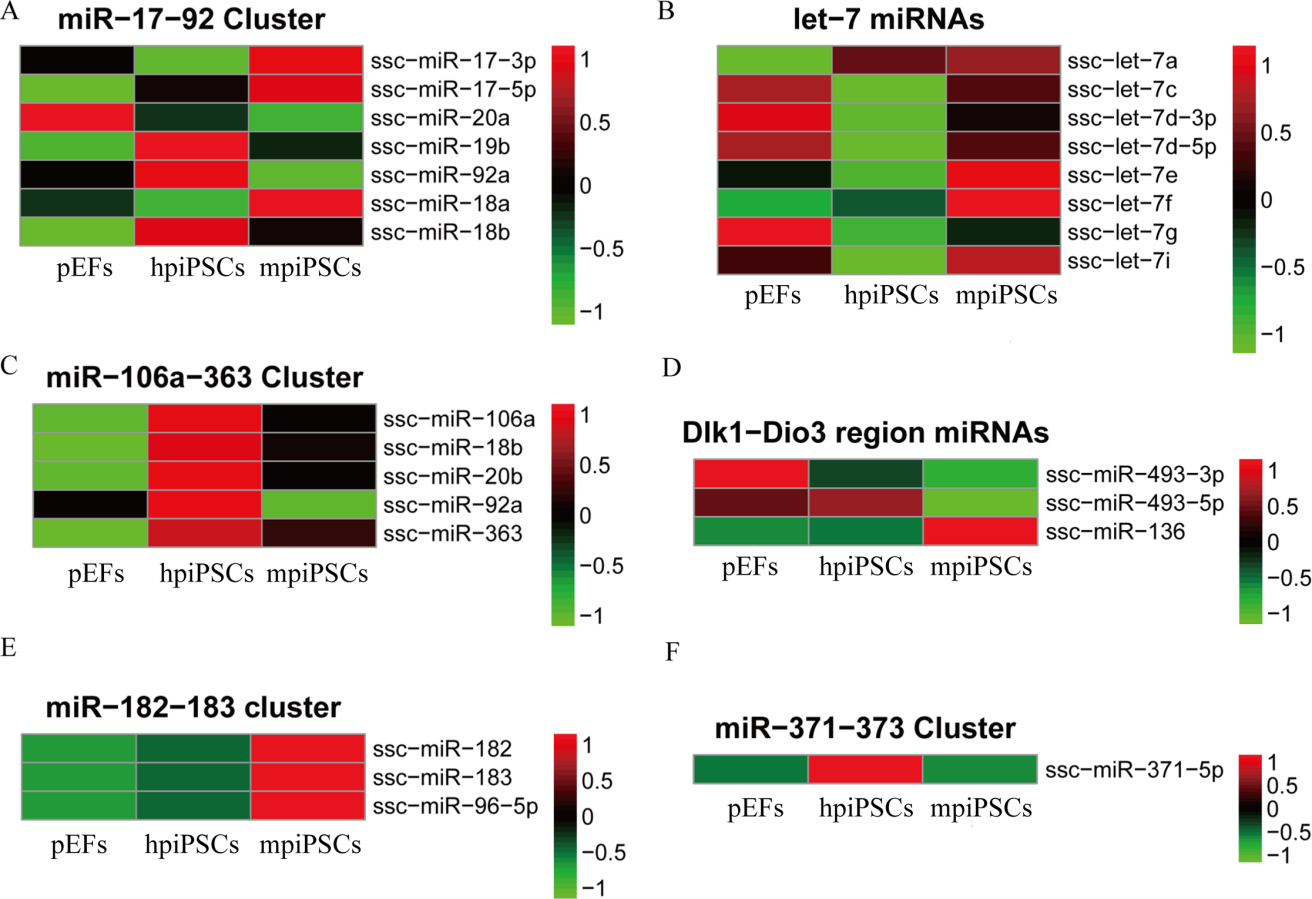
Expression of key miRNA clusters and families in piPSCs and pEFs. (A) The miR-17-92 cluster. (B) Let-7 miRNAs. (C) The miR-106a-363 cluster. (D) The miRNAs in the Dlk1-Dio3 region. (E) The miR-182-183 cluster. (F) The miR-371 cluster.

The miRNAs clusters are very important for the specific expression and function of miRNAs. Thus, we generated heatmaps for several miRNA clusters. In the miR-17-92 cluster, ssc-miR-17 and ssc- miR-18 had higher expression in mpiPSCs compared with the pEFs. However, ssc-miR-19b, ssc-miR-18b and ssc-miR-92 were activated in hpiPSCs compared with pEFs (**Fig 5A**). The expression of the let-7 family was investigated because inhibition of the let-7 family was previously suggested to promote the reprogramming of somatic cells into piPSCs. We found that most members of the let-7 family (except let-7a) were down-regulated in hpiPSCs compared with pEFs (**Fig 5B**). Let-7a was highly expressed in both types of piPSCs but had a low expression level in pEFs. However, the mpiPSCs did not show down-regulation of most let-7 family members, including let-7c, let-7d, let-7e, let-7f, let-7g and let-7i. All the members of the miR-106a-363 cluster were more highly expressed in hpiPSCs compared with mpiPSCs (**Fig 5C**). Of the miRNAs in the porcine Dik1-Dio3 region, there were four annotated miRNAs: ssc-miR-127, ssc-miR-495, ssc-miR-493 and ssc-miR-136. The sequencing studies only detected reads for ssc-miR-493-3p, ssc-miR-493-5p and ssc-miR-136. Ssc-miR-136 was highly expressed in mpiPSCs but it had low expression in hpiPSCs. Ssc-miR-493-5p and ssc-miR-493-3p had lower expression levels in mpiPSCs compared with pEFs (**Fig 5D**). Interestingly, we found that all three miRNAs of the miR-182-183 cluster were up-regulated in mpiPSCs **(Fig 5E**). The porcine ssc-miR-371-5p, which shares a similar seed region as the has-miR-371-373 cluster, was only up-regulated in hpiPSCs (Fig 4F). The expression of key miRNA clusters and families showed that mpiPSCs and hpiPSCs have distinct miRNA signatures.

### A putative porcine miR-302 cluster was predicted by miRdeep2

**Fig 6:**
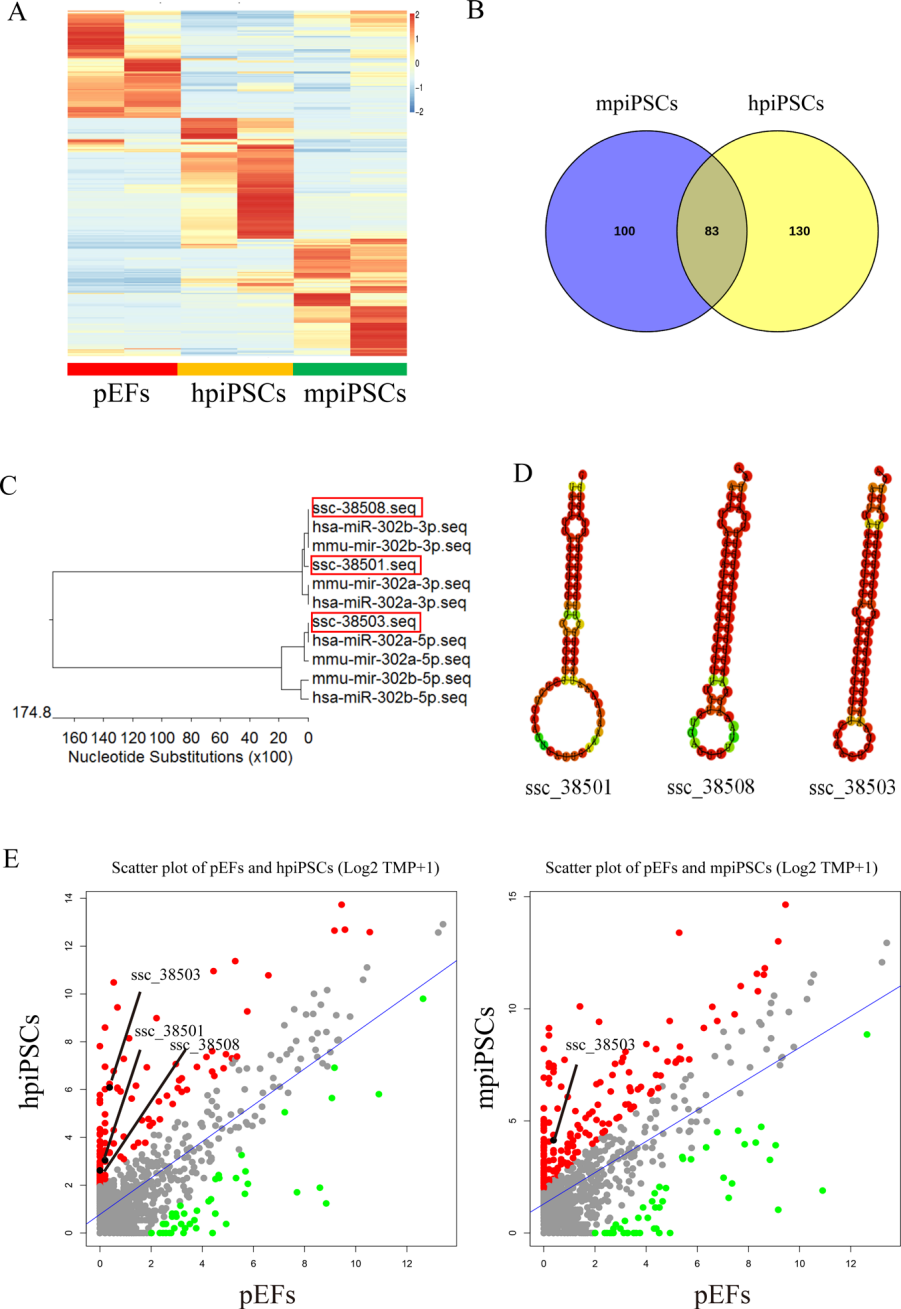
Novel porcine miRNAs predicted by miRdeep2. (A) Heatmap of the differentially expressed novel miRNAs. (B) Venn diagram of the differentially expressed novel miRNAs. (C) BLAST results for ssc_38501, ssc_38503 and ssc_38508 with has-miR-302a/b and mmu-miR-302a/b. (D) Secondary structures of the ssc_38501, ssc_38503 and ssc_38508 pre-miRNAs. (E) Scatter plot of the miRNA expression of piPSCs compared with pEFs. The up-regulated miRNAs are shown as red spots while the down-regulated miRNAs are shown as green spots. The ssc_38501, ssc_38503 and ssc_38508 are shown as black spots.

The number of known porcine miRNAs in the miRNA database (miRBase) is very limited; therefore, we had to use the miRDeep2 software program to predict the novel miRNAs from pigs. We found almost 1014 novel miRNAs using this program. The pEFs, hpiPSCs and mpiPSCs also had different miRNA expression patterns of these novel miRNAs, which was consistent with that of the known mature miRNAs (**Fig 6A**). There were 83 novel miRNAs that were differentially expressed in both hpiPSCs and mpiPSCs compared with pEFs, with 100 novel miRNAs in mpiPSCs and 130 in hpiPSCs, respectively (**Fig 6B** and **S4 File**). There was no porcine miR-302-367 cluster annotation in miRBase v2.0. Of note, we found three novel miRNAs, ssc_38501, ssc_38503 and ssc_38508, which were very similar to has-miR-302a and has-miR-302b in terms of their sequences (**Fig 6C**). Their pre-miRNA sequences had classic stem-loop structures (**Fig 6D**), and all were located in one genomic locus in chromosome 8 (**S2 Table**). However, whereas ssc_38501, ssc_38503 and ssc_38508 were significantly more highly expressed in hpiPSCs compared with pEFs, only ssc_38503 was up-regulated in mpiPSCs compared with pEFs (**Fig 6E**). This indicated that hpiPSCs dominantly expressed the miR-302 cluster.

### Porcine iPSCs-enriched miRNAs promote reprogramming

**Fig 7:**
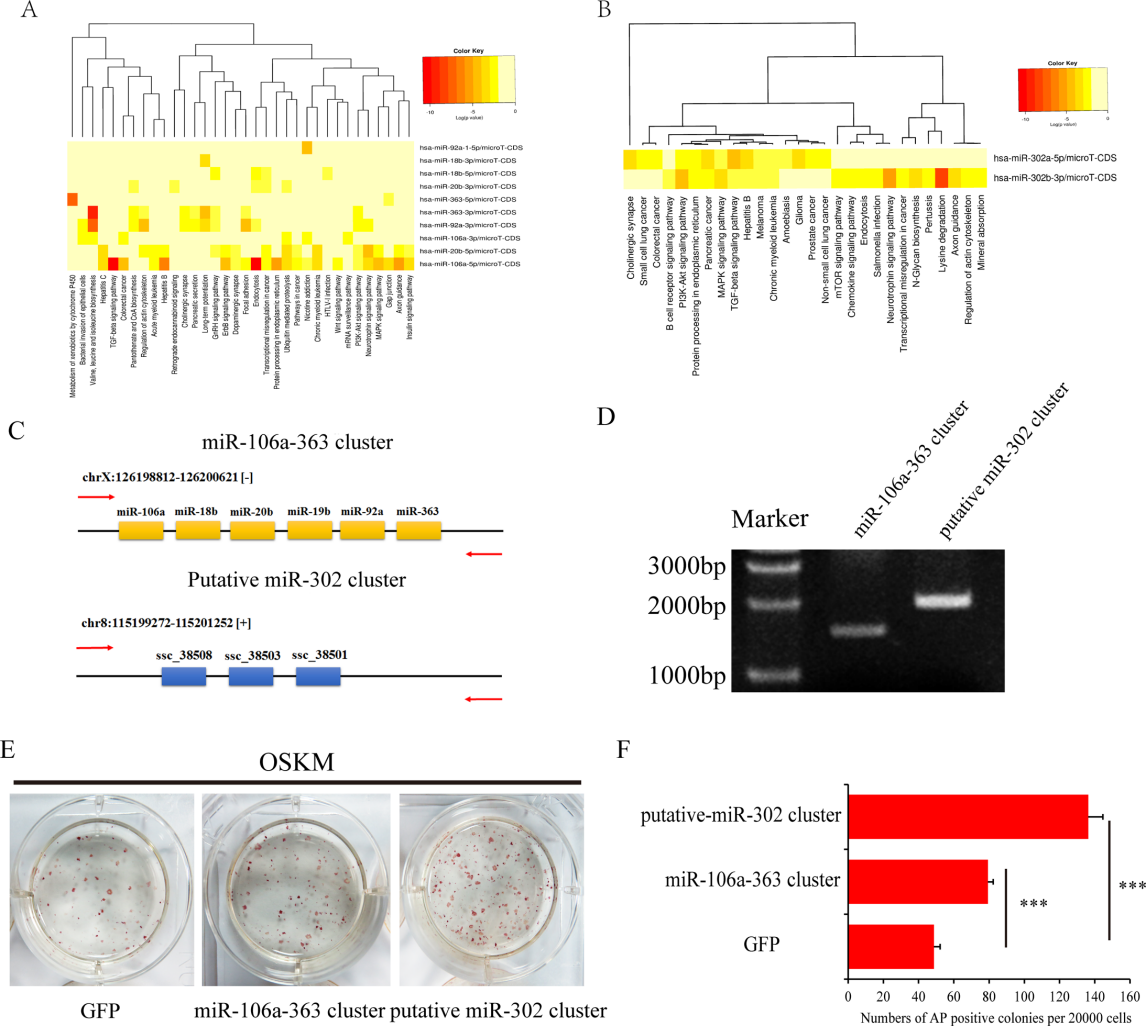
The piPSCs-enriched miRNA cluster promotes the reprogramming of porcine cells. (A) The DIANA miPath program predict the pathways regulated by the miR-106a-363 cluster. Heatmap of the miR-106a-363 cluster versus other pathways, where the miRNAs are clustered together when they exhibited similar pathway-targeting patterns, and the pathways are clustered together by related miRNAs. (B) The DIANA miPath program predicted the pathways regulated by the miR-302 cluster. (C) The PCR region used to clone the genomic locus of the miR-106a-363 cluster and putative miR-302 cluster. (D) Amplification of the genomic region containing the miR-106a-363 cluster and putative miRNA-302 cluster. An approximately 1.5 kb range harboring the whole miRNA-106a-363 cluster and a 2 kb range containing the putative porcine miR-302 cluster were amplified. The marker indicates the DNA ladder. (E) AP staining of the piPSCs colonies. (F) Statistical analysis of AP positive colonies. Mean values ± SD are shown. ***P-value < 0.001.

The miR-106a-363 cluster and miR-302 cluster were both highly expressed in piPSCs compared with pEFs. The DIANA miRPath software predicted that these miRNAs regulate various signaling pathways. The miR-106a-363 cluster regulates ubiquitin-mediated proteolysis, endocytosis, PI3K-Akt signaling pathway, TGF-β signaling pathway and so on (**Fig 7A**). The miR-302 cluster regulates the PI3K-Akt signaling pathway, MAPK signaling pathway, TGF-β signaling pathway, lysine degradation and other processes (**Fig 7B**). It was previously reported that the miR-106a-363 cluster and miR-302 cluster promote reprogramming in mice by negatively regulating the TGF-β signaling pathway [35,36]. In the present study, we cloned the whole genomic loci of the two miRNA clusters into the pMXs vector system (**Fig 7C** and **Fig 7D**). During reprogramming, these miRNA clusters were combined with OSKM to infect pEFs. We observed that the number of AP-positive colonies was highest in the cells transfected with the putative miR-302 cluster group, then in those with the miR-106a-363 cluster group then in the control group **(Fig 7E** and **Fig 7F**). These findings indicate that the miR-106a-363 cluster and putative miR-302 cluster exhibited an improved reprogramming efficiency, which was consistent with the results observed in mouse cells.

## Discussion

MiRNAs play an important role in reprogramming, pluripotency and stem cell fate decisions. Although many investigations have been performed to understand how pluripotency is regulated by miRNAs in both mouse and human pluripotent cells, very limited information has been reported on porcine pluripotent stem cells. We have previously reported that mpiPSCs and hpiPSCs derived under different culture conditions showed different characteristics in terms of their pluripotent markers, incorporation into embryos, metabolism and patterns of gene expression [22]. In the current study, we found that the mpiPSCs and hpiPSCs also had different miRNA expression profiles, which were distinct from those of pEFs.

Compared with pEFs, miRNAs such as ssc-miR-145-5p and ssc-miR-98 were significantly down-regulated in both types of piPSCs. MiR-145 represses the 3’UTR regions of OCT4, SOX2 and KLF4. Increased miR-145 expression inhibits hESC self-renewal, represses the expression of pluripotent genes, and induces the expression of lineage-restricted differentiation. The loss of miR-145 impairs differentiation and increases the levels of OCT4, SOX2 and KLF4 [37]. MiR-98 belongs to the let-7 family and also targets MYC [38]. Inhibition of the let-7 family by Lin 28a, a very important pluripotent factor, improves the efficiency of somatic cell reprogramming [39]. The present findings indicate that the fibroblast-enriched miRNAs, such as ssc-miR-145-5p and ssc-miR-98, may serve as barriers to reprogramming. Repression of these miRNAs appears to be very important for the induction of piPSCs. However, there were 27 DE miRNAs up-regulated in both two types of piPSCs versus pEFs. Among these DE miRNAs, ssc-miR-31 and ssc-miR-1839-5p were selected to confirm the small RNA sequencing results. Ssc-miR-31 was highly expressed in mESCs, as well as in somatic stem cells [40,41]. Ssc-miR-1839-5p have higher expression level than most other DE miRNAs. Conserved miR-1839-5p was also expressed in mESCs [42]. The relative expression of ssc-miR-31 and ssc-miR-1839-5p performed by quantitative stem-loop RT-PCR was consistent with small RNA sequencing.

In a previous study, mouse ESCs, EpiSCs and hESCs were found to express different sets of pluripotency-associated miRNAs, where mouse ESCs dominantly expressed the miR-290-295 cluster and miR-17-92 cluster. However, mouse EpiSCs and hESCs dominantly expressed the miR-302-367 cluster [12]. In our study, the putative porcine miR-302 cluster was highly expressed in both types of piPSCs, but hpiPSCs had a much higher expression level that was similar to that in EpiSCs. The miR-371-373 cluster is specifically expressed in hESCs and is thought to be involved in stem cell maintenance [43]. The porcine miR-371 was only highly expressed in hpiPSCs, which indicated that hpiPSCs resembled hESCs. Ssc-miR-195 and ssc-miR-497 were highly expressed in hpiPSCs and they were also located in the same genome loci in chromosome 12. It reported that has-miR-195-497 cluster was intracellular antagonists of BMP signaling pathway in bone cells in and targeting cell cycle proteins CDK6, CCNE1, CDC25A and CDK4 to regulated cell proliferation in human hepatocellular carcinoma cells [44,45]. BMP signaling pathway is not dispensable of primed state but induced differentiation for hESCs [46,47]. It indicated that ssc-miR-195-497 cluster may maintain hpiPSCs state by regulating cell cycle and BMP signaling pathway.

The miR-182-183 cluster was highly expressed in mpiPSCs. It was previously reported that the miR-182-183 cluster was also highly expressed in mESCs [48]. The miR-182-183 cluster functions in the development of animal sensory organs [49,50]. GSK3β inhibits the expression of miR-96, miR-182 and miR-183 through the β-Catenin/TCF/LEF-1 pathway [51]. We used a GSK3β inhibitor, CHIR99021, to maintain the mpiPSCs, which may explain why the miR-182-183 cluster was highly expressed in the mpiPSCs. The distinct miRNA expression pattern indicated that not only did both types of piPSCs have characteristics of pluripotency but also the hpiPSCs resembled hESCs and the mouse EpiSCs, whereas and mpiPSCs were similar to mESCs. These differences indicated that hpiPSCs and mpiPSCs have distinct mechanisms of post-transcription regulation.

Interestingly, the mpiPSCs were characterized by high expression of miR-217. MiR-217 was up-regulated by TGF-β and was able to activate Akt through the down-regulation of PTEN to increase glomerular mesangial cell survival and hypertrophy [52]. However, miR-217 was found to target NR4A2 in hESCs to block differentiation. NR4A2 was also targeted by has-miR-302d and has-miR-371, which indicates that miR-217 has some regulatory functions in common with other pluripotency-associated miRNAs in hESCs [53]. Ssc-miR-216 was also highly expressed in mpiPSCs. Ssc-miR-216 and ssc-miR-217 were also located in the same genome loci in chromosome 3. Overexpression of has-miR-216a/217 activates the PI3K/Akt and TGF-β signaling pathways by targeting PTEN and SMAD7 in human hepatocellular carcinoma cells [54]. It indicated that ssc-miR-216-217 cluster may participate in regulation TGF-β signaling pathway in mpiPSCs. Ssc-miR-142-5p was also more highly expressed in mpiPSCs than that in hpiPSCs. It is reported that miR-142 is bimodally expressed in mESCs. Low miR-142 expression can respond to differentiation cues [55]. It indicated that ssc-miR-142-5p makes contribution to maintenance pluripotency in mpiPSCs. In the porcine Dlk1-Dio3 locus, most miRNAs were silenced except miR-136, which was highly expressed in the mpiPSCs. In contrast, the miRNAs in the Dlk1-Dio3 imprint region were activated in fully pluripotent mouse stem cells [20]. It was already reported in a previous study that the major imprint genes in the porcine Dlk1-Dio3 region were silenced during porcine fibroblasts reprogramming [56]. This indicates that both types of piPSCs have defects in Dlk-Dio3 locus activation.

This study demonstrated that overexpression of the whole porcine miR-106a-363 cluster and miR-302 cluster combined with OSKM improved the efficiency of piPSCs induction. The miR-106a-363 cluster was demonstrated to share the same or a very similar seed region with the miR-302a-367 cluster members. Overexpression of the miR-106a-363 cluster and miR-302-367 cluster was previously found to increase the efficiency of generating mouse induced pluripotent stem cells using Sox2, Klf4, and Oct4. This was likely because miR-106a and miR-302-367 cluster directly target TGF-β receptor 2 (TGFBR2) and accelerate the mesenchymal-to-epithelial transition (MET) necessary to induce mouse iPSCs [35,36]. TGFBR2 is a part of the TGF-β signaling pathway, which is known to inhibit the MET and serves as a reprogramming barrier [57]. Transfection of has-miR-302a and has-miR-302b miRNA mimics effectively enhanced the porcine reprogramming efficiency and reduced the induction time for piPSCs in the OSKM and OSK induction systems in a previous study [58]. Our present results are in close agreement with those previous findings. The miR-106a-363 cluster and putative miR-302 cluster therefore appear to have conserved functions in promoting the reprogramming efficiency in porcine cells.

Whether hpiPSCs or mpiPSCs as a pluripotent population requires a balance between proliferation and self-renewal. The network of putative target genes and DE miRNAs suggested that hpiPSCs-specific miRNAs regulated p53 signaling pathway, Neurotrophin signaling pathway and cell cycle. P53 induces differentiation of mouse embryonic stem cells by suppressing Nanog expression [59]. Knockdown of p53 was shown to promote human iPSCs generation, which suggested that p53-p21 pathway serves as a barrier in iPSCs generation [60]. Cell cycle regulated by ESCs-specific miRNAs is also very important to keep self-renewal of pluripotent stem cells [61]. These mpiPSCs-specific miRNAs regulated VEGF signaling pathway, apoptosis, Neurotrophin signaling pathway and MAPK signaling pathway. Blocking VEGF signaling pathway is capable of maintaining the mESCs in the undifferentiated state [62]. Antagonizing reprogramming-induced apoptosis in mouse fibroblasts increased the reprogramming efficiency [63]. Inhibiting MAPK pathways by BMP4 supports self-renewal of mESCs [64]. Neurotrophin signaling pathway induced by Neurotrophin-3 promotes differentiation of mouse bone mesenchymal stem cells into neurons [65]. It suggested that specific-miRNAs in hpiPSCs or mpiPSCs may prevent piPSCs neural differentiation by regulation of Neurotrophin signaling pathway. This study identified the putative pathways targeted by these piPSCs-specific miRNAs. These evidences indicated that piPSCs-specific miRNAs regulated corresponding signaling pathways to maintain pluripotency and self-renewal.

**Fig 8:**
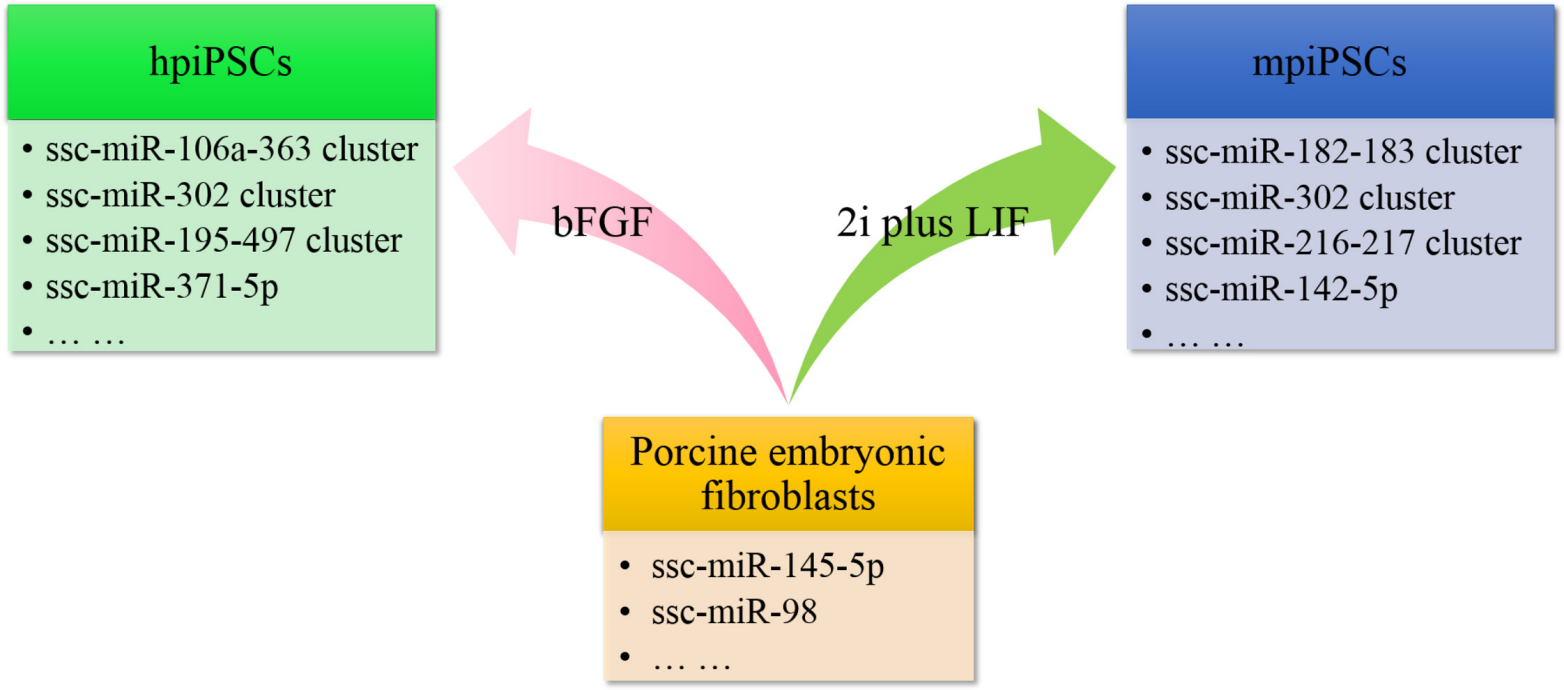
Representative miRNAs and miRNA clusters of pEFs, hpiPSCs and mpiPSCs.

In conclusion, pEFs, hpiPSCs and mpiPSCs showed different miRNA expression patterns. The hpiPSCs and mpiPSCs derived from different culture conditions were both enriched in pluripotency-associated miRNAs but exhibited distinct expression patterns (**Fig 8**). Based on the miRNA expression profiles, the hpiPSCs resembled mouse EpiSCs and hESCs and the mpiPSCs were similar to mouse ESCs. Of note, the piPSCs highly expressing the miR-106a-363 cluster and putative porcine miR-302 cluster had enhanced reprogramming efficiency. Overall, this study presented distinct miRNA expression profiles for two types of piPSCs and provided insights into the mechanisms underlying pluripotency in pigs.

## Supporting Information

S1 Fig (A) TPM values of ssc_38501, ssc_38503 and ssc_38508 in pEFs, mpiPSCs and piPSCs. (B) Results of the quantitative RT-PCR assay of ssc_38503 in pEFs, mpiPSCs and hpiPSCs.
(TIF)

S1 File Sequencing data filter and mapping. (XLSX)

S2 File Differential expressed miRNAs of hpiPSCs versus mpiPSCs. (XLSX)

S3 File Predicted target genes of the miRNAs that were specifically differential expressed in the mpiPSCs and hpiPSCs. (XLSX)

S4 File Differential expression of the novel miRNAs of piPSCs versus pEFs. (XLSX)

S1 Table Primers used for quantitative stem loop RT-PCR of miRNAs and primers used for amplification of miRNA clusters. (DOCX)

S2 Table Genomic location of the putative porcine miR-302 cluster. (DOCX)
