# Supplementary figures and images for: Distinct MicroRNA Expression Signatures of Porcine Induced Pluripotent Stem Cells under Mouse and Human ESC Culture Conditions
Source: PLoS One. 2016 Jul 6;11(7):e0158655. doi: 10.1371/journal.pone.0158655 (PMC4934789; doi:10.1371/journal.pone.0158655)

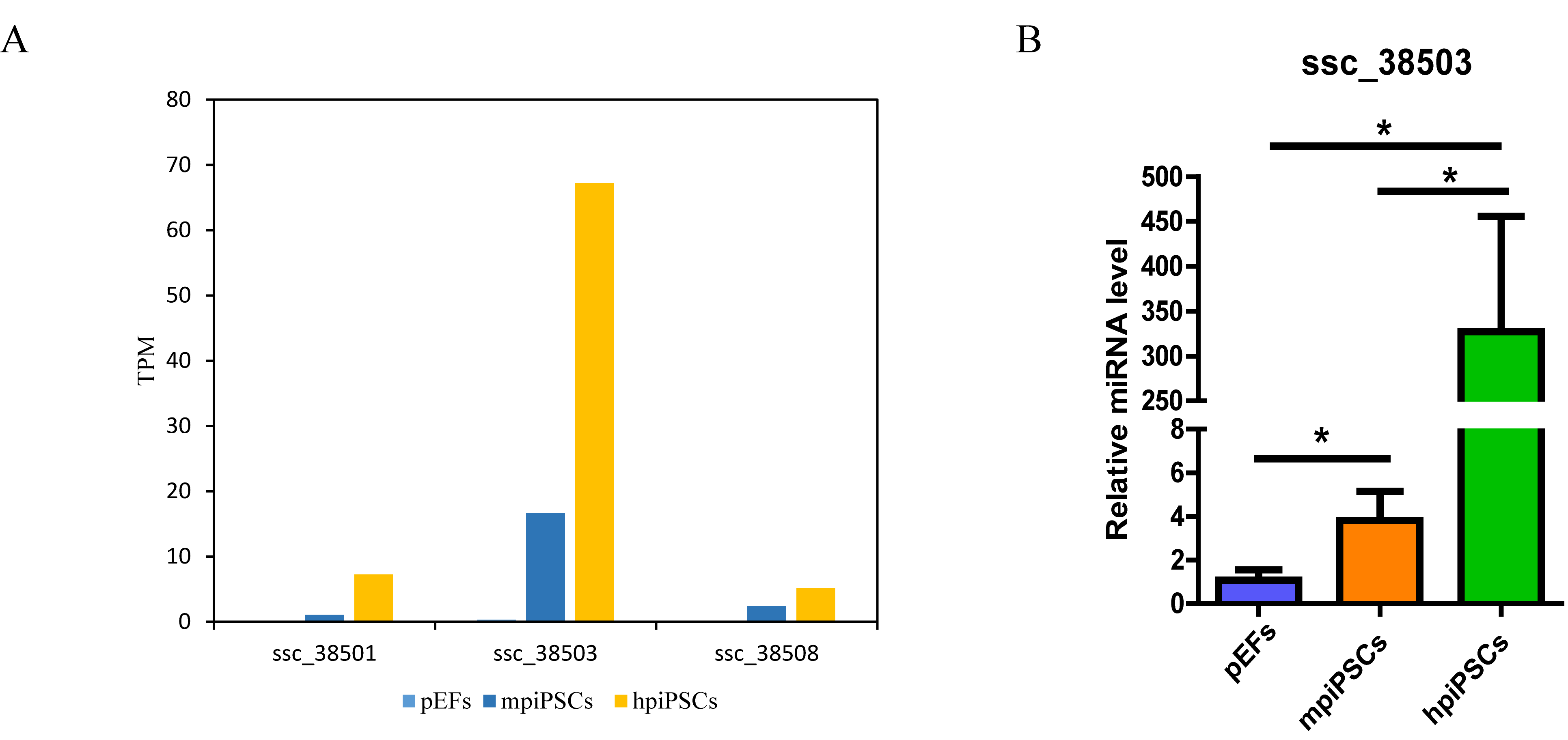

Supplement: S1 Fig — (A) TPM values of ssc_38501, ssc_38503 and ssc_38508 in pEFs, mpiPSCs and piPSCs. (B) Results of the quantitative RT-PCR assay of ssc_38503 in pEFs, mpiPSCs and hpiPSCs. (TIF) [file pone.0158655.s001.tif]
