# Supplementary material for: Distinct MicroRNA Expression Signatures of Porcine Induced Pluripotent Stem Cells under Mouse and Human ESC Culture Conditions
Source: PLoS One. 2016 Jul 6;11(7):e0158655. doi: 10.1371/journal.pone.0158655 (PMC4934789; doi:10.1371/journal.pone.0158655)
Supplement: S1 Table — (DOCX) [file pone.0158655.s006.docx]

**Primers for quantitative RT-PCR**

| **miRNA** | **Mature miRNA sequence** | **RT primer** | **Forward primer** | **Reverse primer** |
| --- | --- | --- | --- | --- |
| **miR-145** | GUCCAGUUUUCCCAGGAAUCCCUU | CTCAACTGGTGTCGTGGAGTCGGCAATTCAGTTGAGAGGGATTC | ACACTCCAGCTGGGGTCCAGTTTTCCCAGGA | CTCAACTGGTGTCGTGGAGTC |
| **miR-98** | UGAGGUAGUAAGUUGUAUUGUU | CTCAACTGGTGTCGTGGAGTCGGCAATTCAGTTGAGAACAATAC | ACACTCCAGCTGGGTGAGGTAGTAAGTTGT | CTCAACTGGTGTCGTGGAGTC |
| **miR-1839-5p** | AAGGUAGAUAGAACAGGUCUUG | CTCAACTGGTGTCGTGGAGTCGGCAATTCAGTTGAGCAAGACCT | ACACTCCAGCTGGGAAGGTAGATAGAACAG | CTCAACTGGTGTCGTGGAGTC |
| **miR-31** | AGGCAAGAUGCUGGCAUAGCUG | CTCAACTGGTGTCGTGGAGTCGGCAATTCAGTTGAGAGCTATGC | ACACTCCAGCTGGGAGGCAAGATGCTGGC | CTCAACTGGTGTCGTGGAGTC |
| **miR-363** | AAUUGCACGGUAUCCAUCUGUAA | CTCAACTGGTGTCGTGGAGTCGGCAATTCAGTTGAGTACAGATG | ACACTCCAGCTGGGAATTGCACGGTATCCA | CTCAACTGGTGTCGTGGAGTC |
| **miR-106a** | AAAAGUGCUUACAGUGCAGGUAGC | CTCAACTGGTGTCGTGGAGTCGGCAATTCAGTTGAGCTACCTGC | ACACTCCAGCTGGGAAAAGTGCTTACAGTGC | CTCAACTGGTGTCGTGGAGTC |
| **miR-371-5p** | ACUCAAACUGUGGGGGCACUUU | CTCAACTGGTGTCGTGGAGTCGGCAATTCAGTTGAGAAAGTGCC | ACACTCCAGCTGGGACTCAAACTGTG | CTCAACTGGTGTCGTGGAGTC |
| **miR-183** | UAUGGCACUGGUAGAAUUCACUG | CTCAACTGGTGTCGTGGAGTCGGCAATTCAGTTGAGCAGTGAAT | ACACTCCAGCTGGGTATGGCACTGGTAGAAT | CTCAACTGGTGTCGTGGAGTC |
| **miR-182** | UUUGGCAAUGGUAGAACUCACACU | CTCAACTGGTGTCGTGGAGTCGGCAATTCAGTTGAGTGTGAGTT | ACACTCCAGCTGGGTTTGGCAATGGTAGAA | CTCAACTGGTGTCGTGGAGTC |
| **miR-217** | UACUGCAUCAGGAACUGAUUGGAU | CTCAACTGGTGTCGTGGAGTCGGCAATTCAGTTGAGTCCAATCA | ACACTCCAGCTGGGTACTGCATCAGGAACTG | CTCAACTGGTGTCGTGGAGTC |
| **ssc_38503** | ACUUAAACGUGGAUGUACUUGCU | CTCAACTGGTGTCGTGGAGTCGGCAATTCAGTTGAGTCACCAAA | ACACTCCAGCTGGGTAAGTGCTTCCATGTTT | CTCAACTGGTGTCGTGGAGTC |
| **U6** |  | CGCTTCACGAATTTGCGTGTCAT | GCTTCGGCAGCACATATACTAAAAT | CGCTTCACGAATTTGCGTGTCAT |

**Primers for cloning miRNA clusters**

|  |  | **Primer sequence (5‘-3)’** | **Product size (bp)** |
| --- | --- | --- | --- |
| **miR-302-F** | Forward primer | CCTTAATTAAGCAAAGTTCCCTTTGCCCCA | 1944 |
| **miR-302-R** | Reverse primer | CCCTCGAGAGTAGAGGTTACCGAACCCCA |  |
| **miR-106-F** | Forward primer | CCTTAATTAAACCCGCACATTTTTCTGGGA | 1449 |
| **miR-106-R** | Reverse primer | CCCTCGAGCAGGAACAATCCCGTCCGAT |  |

Red words mean the restriction endonuclease site
