# Supplementary material for: Distinct MicroRNA Expression Signatures of Porcine Induced Pluripotent Stem Cells under Mouse and Human ESC Culture Conditions
Source: PLoS One. 2016 Jul 6;11(7):e0158655. doi: 10.1371/journal.pone.0158655 (PMC4934789; doi:10.1371/journal.pone.0158655)
Supplement: S2 Table — (DOCX) [file pone.0158655.s007.docx]

Genome loci and sequences of putative miR-302 cluster

|  | **Mature sequence** | **Precursor sequence** | **Genome location** |
| --- | --- | --- | --- |
| Ssc_38501 | UAAGUGCUUCCAUGUUUUAGUGC | UACUUUAACAUGGAGCCACUUGCUGUGAAUUGAUUGAAAAAAAAAUAAGUGCUUCCAUGUUUUAGUGC | 8:115200553-115200621:+ |
| Ssc_38503 | ACUUAAACGUGGAUGUACUUGCU | ACUUAAACGUGGAUGUACUUGCUUUCAAACUCUAAAAGUAAGUGCUUCCAUGUUUUAGUGA | 8:115200389-115200450:+ |
| Ssc_38508 | UAAGUGCUUCCAUGUUUUAGUAG | ACUUUAACAUGGGAGUGCUUUCUGUGAGUUUUAAAGUAAGUGCUUCCAUGUUUUAGUAG | 8:115200080-115200139:+ |
